# News analysis

**DOI:** 10.1136/tc.2009.034454

**Published:** 2009-11-17

## NEW ZEALAND: INDUSTRY FACES GRILLING OVER MAORIS

All articles written by David Simpson unless otherwise attributed. Ideas and items for News Analysis should be sent to: d.simpson@iath.org

In which country might one hear the most refreshingly straight talk about the tobacco industry in the national legislature? There may be no easy, single answer, but recent experience suggests that New Zealand must be a contender.

While the majority of New Zealanders have been reducing their tobacco consumption for several decades, the indigenous Maori people still have alarmingly high smoking rates. New data announced by the health ministry in September showed that 49.3 per cent of Maori women and 41.5 per cent of Maori men still smoke, compared to just over a fifth of the adult population as a whole. Like many countries with a relatively long history of public education about tobacco, it is among the lower income, more vulnerable minority population where tobacco companies see their greatest hopes of sustaining sales.

Not surprisingly, then, Mr Hone Harawira, a member of parliament for the Maori Party, wants tobacco company executives to be summoned personally to face questions at an inquiry by the Maori Affairs parliamentary select committee into the impact of tobacco use on Maori people. The committee would talk to everybody concerned, he said, before it got to the tobacco companies, implying that he was determined that the fullest powers available to the parliament's Speaker be used to force the New Zealand-based chairpersons and chief executives, not just the public relations “spin doctors”, to be involved.

Mr Harawira made it plain that he expected this to be an uncomfortable experience for those involved. Just in case anyone missed any slight nuance of meaning in his announcement, he added: “to be brutally frank I’d like to lynch these bastards... This is a war against people who kill New Zealanders ... I don’t particularly give a shit about what they say [in their defence].”

## URUGUAY: NEW HEALTH WARNINGS

Uruguay is set to have the largest health warnings in the world. A new set of six graphic pack warnings, each covering 80 per cent of the front and back of the pack, was finalised in September. They will have to be in place by 1 March 2010, though the warnings they replace, which covered 50 per cent of the pack, were ordered to be expanded to 80 per cent by December. The two large sides of the pack are not the only place where Uruguayan smokers will see health warning information: a statement will also be required on the side of tobacco packages, in black on white, to the effect that the product inside contains nicotine, tar and carbon monoxide.

The bold assurance with which a relatively small country in Latin America has ordered yet another set of warnings—the fourth since 2006—is a mark of just how far this aspect of tobacco control has come in a relatively short time, as well as of the Uruguayan government’s admirable determination to place appropriate labels on a uniquely dangerous product. The bloody fights waged in countries such as Canada, a pioneer of modern graphic warnings, seem a very long time ago. Absurd arguments and desperate threats may still be used by the tobacco industry—in Canada, for example, they gave straight-faced assurances that more than a few colours were technically impossible, while threatening to take their package printing work to the USA—but now that the great graphic warnings race is well and truly off the starting blocks, we should see progressively less of such nonsense, and progressively more creative and effective ways of showing just what is contained in those once alluring packs.

**Figure:**
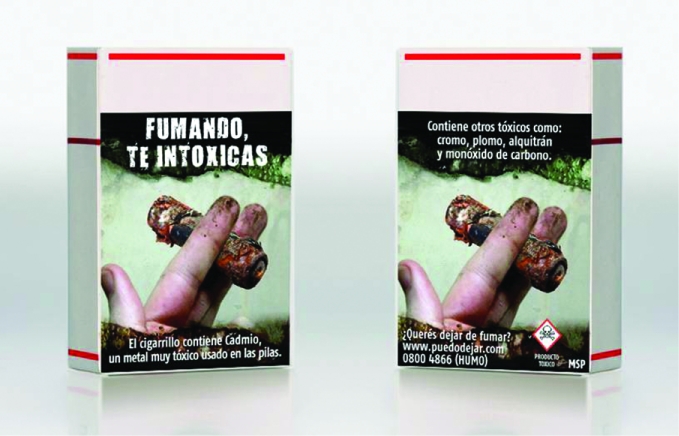
Uruguay: the back and front of one of the new Uruguayan pack health warnings, focusing on the little known presence of toxic chemicals such as lead and cadmium in cigarette smoke.

## KAZAKHSTAN: PUBLIC SMOKING BAN

Like Bulgaria (see page 429), just a few years ago Kazakhstan would not have been expected to turn up on a list of countries likely to ban smoking in all public places. But as the health ministry said when announcing such a move in September, the central Asian country is now following the recommendations of the World Health Organization, according to whose data more than 30,000 people die every year in Kazakhstan from smoking. Sports stadiums and public transport facilities were already smoke-free, but Kazakh bars and nightclubs, and all other remaining public areas not previously covered by the ban, have now been forced to go smoke-free. At the same time, the age at which people may be sold alcoholic drinks has been raised from eighteen to 21, in view of the country's significant problem of alcoholism.

## RUSSIA: WHAT IS JTI PLAYING AT?

The acting profession and the tobacco industry have shared a long and infamous history, promoting the fiction that smoking is glamorous. While campaigns, including Smoke Free Movies, have exposed the industry’s extensive use of tactics such as paid endorsement and product placement, the linking of smoking with aspirational lifestyles remains a key strategy.

This is evident in a casting call this autumn for jobbing British actors by Eyecandy Model and Promotions Agency for a job to help launch a new brand of cigarette in Russia. But the call is not for an acting part in a commercial film. Regulation of tobacco advertising has progressively tightened in Russia since 2002, with restrictions on television and radio, and a ban on outdoor tobacco advertising since 2007. New legislation is currently making its way through the Russian parliament which would make the country compliant with FCTC stipulations for a comprehensive ban on tobacco advertising, promotion and sponsorship. According to Euromonitor, these restrictions have made it extremely difficult to launch new brands in Russia, currently the third largest cigarette market in the world by volume.

Tobacco companies in Russia have therefore shifted increasingly to indirect marketing methods. The casting call for a “good looking” British actor aged 25-48, with “well groomed hands”, is a good example. The 8-week job takes the now familiar distribution of free samples in bars even further by requiring the actor to play the part of a “vitolier”, a seemingly made up word meaning “tobacco expert”, which is likened to the respected position of sommelier, a master of all aspects of fine wines. For £125 ($204) per day plus £15 ($24) expenses, the actor will visit restaurants, with an interpreter, to play out the following scenario on unsuspecting members of the public.

A script outline dictates the essential shape of each visit, starting with the “vitolier” and interpreter entering a restaurant and taking a seat at the bar, looking for a table with smokers, sending over a waiter with a note in which “a British tobacco expert from London” asks to talk to them. After introducing himself, the actor says he has been invited by  Japan Tobacco International (JTI) to visit their country and that he is “really interested in the tastes of Russian smokers.” He is then to converse with them about their tastes and preferences regarding premium cigarettes, also telling them about “the English tobacco traditions”, mentioning Sobranie cigarettes and recommending the new Sobranie product. Finally, “He tells them about the tones of premium tastes… the latest trends in contemporary art, and recommends the current art-project of Sobranie in Moscow.”

Sobranie of London, established in 1879, is one of the oldest tobacco brands in the world. Originally handmade and supplied to royal courts across Europe, today Sobranie brands (including Black Russian) are produced by JTI and are among the most expensive in Russia. The company’s aim is to encourage smokers to “trade up”, from lower priced cigarettes to higher priced brands. To promote its high-end image, Sobranie also sponsors the Millionaire Fair Moscow, “a leading exhibition of luxury, despite and contrary to the world financial crisis”. The casting of a British actor to play the part of a “vitolier”, dressed in a suit and armed with a “frank, straightforward, honest, and pleasant smile”, is part of that strategy.

What is perhaps most remarkable is how out of touch the whole campaign is with the economic and social realities of life for most Russians today. Amid global recession, the Russian economy has been hard hit by the decline in world commodity prices. Unemployment reached 8.5 per cent in October and the official inflation rate for 2009 is running at 13 per cent. These recent problems have worsened longer term declines in living standards since the mid 1990s. Male life expectancy has plummeted to less than 59 years in 2008 (compared to 72 years for females), well below the 74 years of men in western European countries such as France and Germany. While staggering levels of alcohol consumption have a lot to answer for, smoking is also taking a major toll. With male smoking rates at 61 per cent, the number of reported cases of lung cancer has increased by 63 per cent over the last ten years. Smoking is cited as the main cause for 52 per cent of all cancer cases, the biggest cause of premature deaths alongside heart disease.

With most Russians struggling to earn a decent living, JTI’s attempt to launch a new luxury brand of cigarette really does belong in the realm of fantasy. The whole campaign has an air of fiddling while Rome burns. One must also ask whether tobacco advertising executives really are that out of touch with reality. Or is this just another example of the creativity of the industry in seeking to circumvent stronger regulation? JTI claims that it “believes that appropriate regulation of tobacco is both necessary and right, in the interest of public health. We believe that the WHO, sovereign governments, non-government organizations and the tobacco industry should all work together to resolve these issues.” If the company really believes this, perhaps it should thus stop play-acting and tell the truth.

KELLEY LEE

London School of Hygiene & Tropical Medicine, UK

kelley.lee@lshtm.ac.uk

## UK: SMOKER'S LUNGS KILL TRANSPLANT MAN

A coroner’s court examining a British soldier’s death from lung cancer in 2008 heard recently that the 31-year-old man had received a bilateral lung transplant the previous year after being diagnosed with an incurable lung condition. Immunosuppressive drugs prescribed to help his body accept the new lungs, which had an undetected tumour, simply increased the speed of its growth. On investigation, it was revealed that he had received the lungs of a smoker of 30 to 50 hand-rolled cigarettes per day.

While most people know that on average, smokers die younger, often from serious diseases caused by their smoking, it came as a shock to realise how many of their bodies were being recycled for transplantation. In 2007, 22 per cent of British adults aged 16 and over smoked and a further 27 per cent were ex-smokers; so it is not surprising that 51 per cent of all organs for transplants originate from smokers. The transplanting hospital said that the soldier’s case was extremely rare, in an area of great need, with half as many patients as receive a lung transplant dying while still on the waiting list for one; and if the hospital did not use the lungs of smokers, the number of lung transplants would be significantly lower.

## CHINA: EXPO REJECTS TOBACCO FUNDS

Organisers of the 2010 Shanghai World Expo recently responded to concern and rejected a 200 million yuan (US$29 million) donation from the Shanghai tobacco company in order to observe the promise of a “healthy and smoke-free Expo”. The announcement came in response to several weeks of heated debate around China in July on the legitimacy of allowing tobacco promotion and sponsorship in a public event like the World Expo.

The debate was partly triggered by an earlier suggestion from a group of Chinese health experts that Expo organisers should reject the donation, which would have been a “public showcase of tobacco advertising” and a “violation of international treaties”. They cited Article 13 of the World Health Organization’s Framework Convention on Tobacco Control (FCTC), under which parties are obliged to undertake a comprehensive ban on tobacco advertising, promotion and sponsorship, at both domestic and international levels. China, the world’s largest tobacco producer and consumer, signed the FCTC in 2003 and committed to ending all types of tobacco advertising and promotion by 2011, and to making workplaces totally smoke-free.

Shanghai Tobacco, which produces China’s major cigarette brands including Panda and Chung Hua, donated 200 million yuan in May to help build the China Pavilion, which is expected to cost 1.5 billion yuan.

The decision by the World Expo authorities to refuse tobacco funding was praised by the World Health Organization. However, health experts say that an even bigger challenge is now before the Shanghai Municipal Government, as it considers how far to go in protecting the city’s population of 17 million people by requiring workplaces and public places to be smoke-free from 2010.

**Figure:**
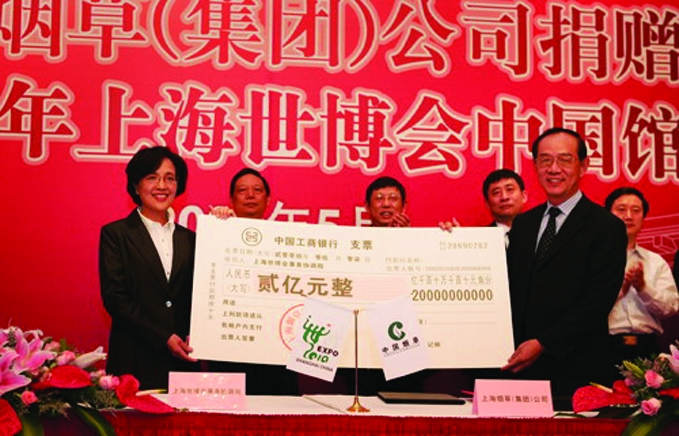
China: Shanghai Expo organisers initially accepted a 200 million yuan (US$29m) cheque from Shanghai Tobacco, which they later rejected.

## THAILAND: WIPING OUT THE LAST ADS

Thailand enacted laws prohibiting all forms of advertising from April 1992 and banning misleading descriptors from March 2006. The law mandating pictorial health warnings, occupying 50 per cent of both principal cigarette pack surfaces, became effective from March 2005. However, despite these stringent regulations, transnational tobacco companies sneakily placed promotional gimmicks on the remaining 50 per cent of pack surfaces on their imported cigarettes, not only promoting the cigarettes, but also effectively ruining the visual impact of the health warnings.

In March 2009, it was found that packages of Philip Morris (PM) brand L&M Select bore such promotional items in the form of colour pictures of tobacco leaves and a peeling filter, with the texts, “A premium smooth taste with lasting flavour. Blended to perfection.” and “A premium cool sensation with lasting flavor. Chilled to perfection.”

As these were considered violations of the Thai laws, PM Thailand Ltd was notified by the tobacco control section of the government’s Department of Disease Control (DDC) and asked to remove all the offending packages from sales outlets. The company asked for and was granted permission to obliterate the illegal pictures and wordings by placing a white paper cover over them.

Not long afterwards, British American Tobacco asked DDC to consider a model prepared by the company for its Pall Mall cigarette packs. The design contained a phrase promoting the product, with a logo stating, “Sun Ripened Tobacco” and the accompanying phrases, “Naurally Sun Ripened Tobacco” and, “More Taste.” The company also enclosed a photocopy of a document from the Department of Intellectual Property accepting BAT’s request to register this as a trademark.

In a decisive blow for health, the proposal was turned down by DDC—shutting the door on a promotion that covered part of the health warning on the cigarette package.

SUNIDA PREECHAWONG

Chulalongkorn University, Thailand

sunida.p@chula.ac.th

**Figure:**
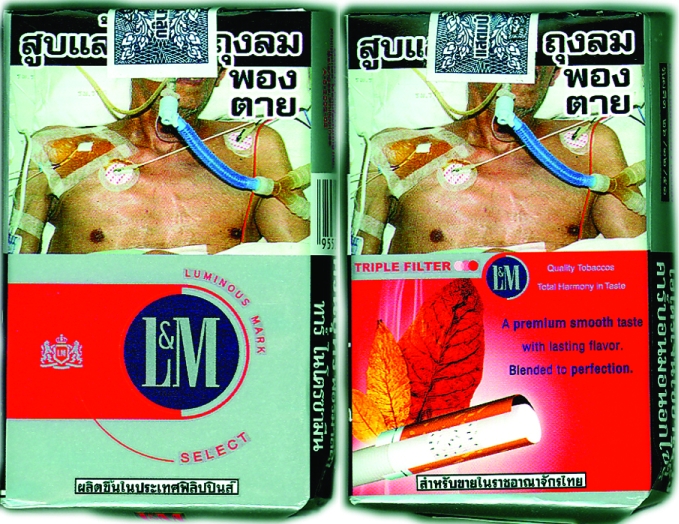


**Figure:**
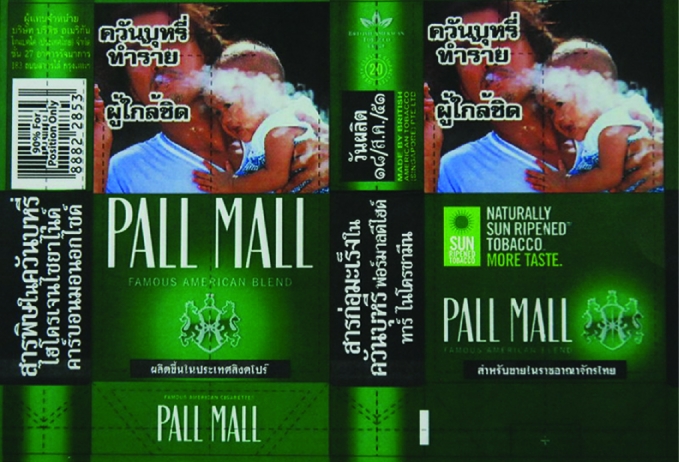
Thailand: companies using promotional devices partly covering the health warnings on cigarette packs to get round Thai advertising and health warning regulations have been ordered to stop using them.

## BULGARIA: TAX RISE TO CURB SMOKING

Having previously had little or no effective tobacco control policies, Bulgaria sprang into action recently when its finance minister not only announced a 43 per cent rise in tobacco excise tax to be applied from next year, but stated plainly why the new government, elected in July, was taking the measure. While it will raise much needed revenue, the minister emphasised that it was primarily a health measure.

Bulgaria is the second heaviest smoking country in Europe, after Greece, partly influenced by its former position as the most important tobacco supplier within the former socialist bloc of central and eastern European states. More than half of men and more than a third of women are smokers, with the population suffering correspondingly high rates of tobacco induced disease. The government is also to ban smoking in all public spaces from June next year. For those whose experience of Bulgarian public places is of some of the smokiest in the world, the implementation of this major change will be of particular interest.

## INDIA: COUNTERING THE IMPACT OF PACK WARNINGS

A new type of product, already familiar in other countries, was launched onto the Indian market recently and is now readily available at easily accessible tobacco outlets at all the markets which young people frequent. The product is called a “smokeshirt”, a name that might suggest that it is made of cloth; however, it is an attractive, sophisticated cover for a cigarette packet. Creatively designed pamphlets promoting smokeshirts are being distributed in up-market areas, apparently targeting Indian young women and girls. They give detailed information about smokeshirts, positioning them as a stylish accessory with designs to suit everyone’s taste. They are available in a variety of top grade textiles, the leaflets say, from stripes and polka dots to leopard skin print, to match one’s clothes and suitable for any occasion.

Currently smokeshirts are being imported from Germany. Made by a company called Lifestyle and Fashion, they were invented by two brothers, Joerg and Michael Knobloch. Their aim was to enable smokers to cover up the graphic health warnings on cigarette packets that are now being adopted in many countries. The Knobloch brothers, who describe the smokeshirt as a “lifestyle product”, reportedly conducted extensive research of patent offices around the world to ensure that they were launching a unique product, as well as selecting product features and pricing based on market research in Germany. They also researched pack sizes of cigarettes around the world to determine the dimensions of their product. A design team then produced a range of smokeshirt designs.

India delayed the implementation of pictorial warnings time and again from 2006, due to pressure from the tobacco industry, but they were finally enforced on 31 May 2009. The launch of smokeshirts in India, just a few months before the pictorial health warnings were implemented, is clearly a way of countering the impact of the new warnings. Along with the implementation of pictorial warnings, India’s new regulations prohibit the sale of any product which can be used to cover or obscure the warnings. Thus, with the new regulations coming into force, the sale of smokeshirts has become illegal in India, but they continue to be on the market. Smokeshirts also have a complementary neckband, whose use makes the smokeshirts easily accessible and visible, making it unnecessary to keep them in a purse or pocket.

Apart from India, smokeshirts are available in European clothing and design shops from Portugal to Hungary and in many locations in China. A statement from the Knobloch brothers about their product reads, “Our retail partners aren’t taking any risks because our products practically sell themselves in no time at all, and they offer excellent value for money. We receive follow-up orders all the time, and we are able to fill them immediately, because it’s always our policy to overproduce during the first manufacturing run. We see this as an important service we offer our retail partners.”

As increasing numbers of countries add the requirement for pictorial health warnings to their tobacco control laws, they will need to address the likely emergence of smokeshirts to counter the effect of the warnings. They will not only need to legislate specifically against products intended to cover up the warnings, but, unlike India, show that they mean business by acting quickly and decisively to enforce this and all other tobacco control regulations.

PRIYANKA DAHIYA & MONIKA ARORA

HRIDAY (Health Related Information Dissemination Amongst Youth), India

monika@hriday-shan.org

**Figure:**
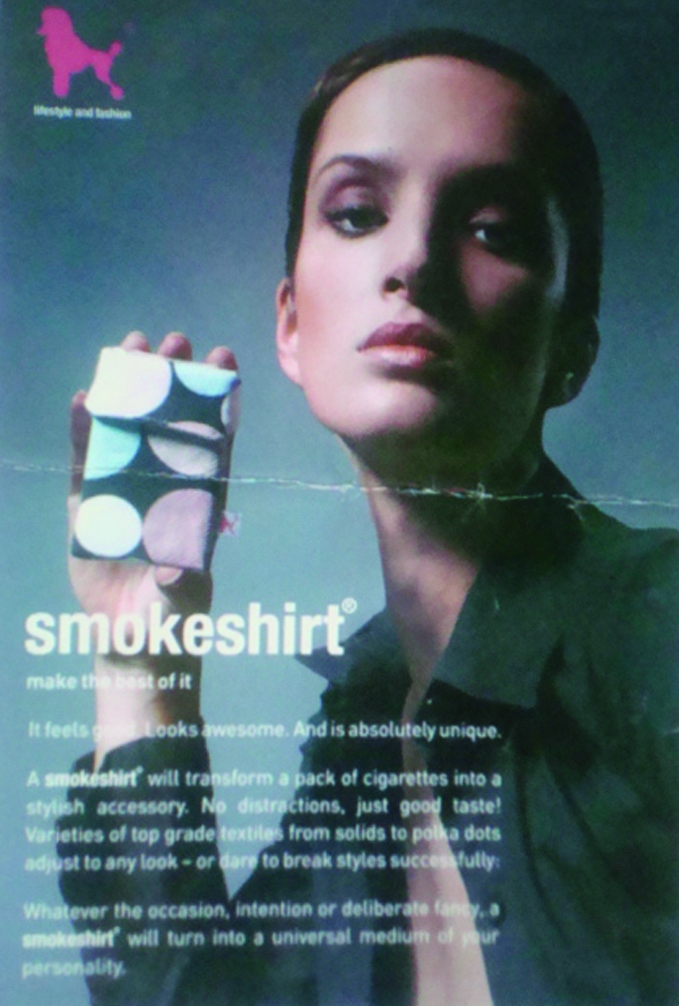
India: a leaflet promoting the Smokeshirt cigarette pack holder.

**Figure:**
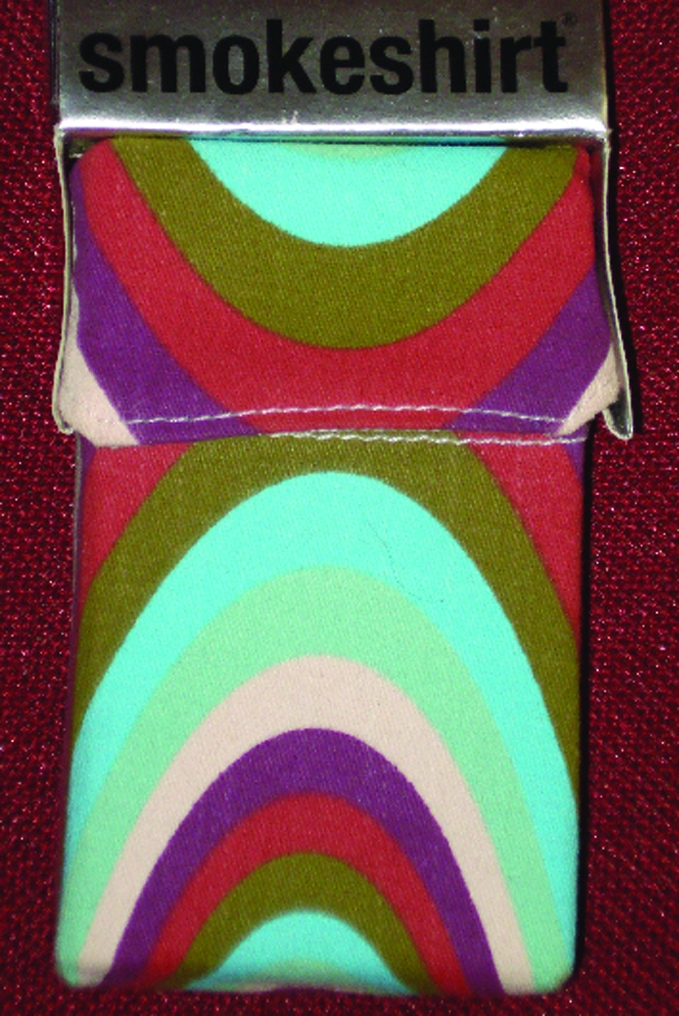
India: a Smokeshirt pack holder.

## MICHAEL RUSSELL

The distinguished research scientist Michael Russell, whose work was so important to understanding why people smoke and how they can be assisted to stop, has died aged 77. Born in Capetown, South Africa, he studied medicine in the United Kingdom, at Oxford and Guy's Hospital, London, returning to South Africa and working as a junior doctor at Groote Schuur Hospital. After deciding to specialise in psychiatry, he and his wife, Audrey, both deeply unhappy with apartheid, returned to London to train at the Maudsley hospital. Working in Griffith Edward’s Addiction Research Unit at the Institute of Psychiatry, he chose smoking as his research thesis topic and in 1971 published a seminal paper concluding that nicotine was the motive force behind smoking.

Recognising the need to measure smoke intake accurately, Russell recruited biochemist Colin Feyerabend to develop a method for measuring blood nicotine. By 1974 he could quantify intake with precision and even measure non-smokers’ exposure from passive smoking, one of many “firsts” in the field. As his understanding grew of smokers' self-titration to maintain nicotine levels, he was also one of the first (at least outside the tobacco industry) to understand how lower emission cigarettes were flawed.

It was Mike Russell who persuaded a pharmaceutical company to produce the world's first nicotine chewing gum, designing a randomised trial to test its efficacy at the Maudsley clinic, as well as later co-developing nicotine nasal spray. His work thus laid the foundations of the nicotine replacement industry.

In addition to the full spectrum of tobacco pharmacology, Russell's research also covered wider aspects of applied cessation. He showed, for example, how the mere completion of a questionnaire about smoking by patients waiting to see a family doctor led to a measurable long-term reduction in their consumption, with brief advice from the doctor yielding around five per cent one year abstinence. He was a familiar figure at tobacco control meetings, where his sometimes fiery defence of his views contrasted with kind and caring support to successive directors of Action on Smoking and Health and other tobacco control workers. Russell was appointed Professor of Addiction in 1986.

He was the recipient of the Alton Ochsner Award, an international prize for outstanding research on tobacco and health, in 1996, and the Ferno Award of the Society for Research on Nicotine and Tobacco in 1998. Having returned to independent South Africa, Michael Russell's last few years of retirement in Capetown were marred by Alzheimer’s disease. He is survived by Audrey and his sons James and Nicholas.

**Figure:**
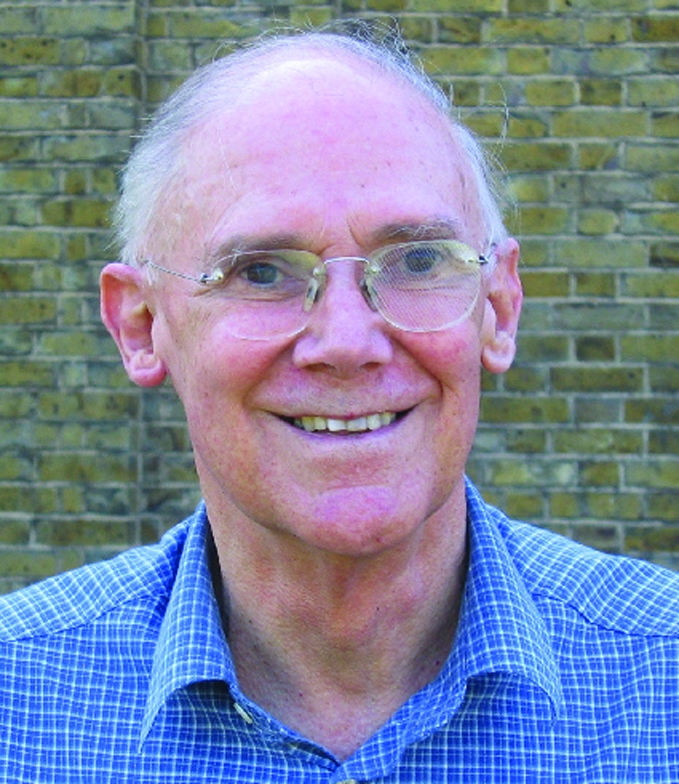
Professor Michael AH Russell, 9 March 1932 to 16 July 2009.

